# Prevalence and persistence of cost-related medication non-adherence before and during the COVID-19 pandemic among medicare patients at high risk of hospitalization

**DOI:** 10.1371/journal.pone.0289608

**Published:** 2023-08-29

**Authors:** James X. Zhang, David O. Meltzer

**Affiliations:** 1 Department of Medicine, The University of Chicago, Chicago, Illinois, United States of America; 2 Harris School of Public Policy, The University of Chicago, Chicago, Illinois, United States of America; 3 Department of Economics, The University of Chicago, Chicago, Illinois, United States of America; Sungkyunkwan University School of Social Sciences, REPUBLIC OF KOREA

## Abstract

**Objective:**

To study cost-related medication non-adherence (CRN) for a 30-month period before and during the COVID-19 pandemic using a sample of Medicare patients at high risk of hospitalization.

**Design:**

A novel data set of quarterly surveys of CRN was used to evaluate CRN before and during the COVID-19 pandemic. Generalized Estimating Equation (GEE) analyses were conducted to evaluate the adjusted coefficients of change in CRN behaviors controlling for socio-demographic and health characteristics.

**Participants:**

Six hundred seventy-seven Medicare beneficiaries at high risk of hospitalization who were alive on January 1, 2020 and followed up through quarterly surveys on CRN for 30 months before and during the COVID-19 pandemic.

**Main outcomes and measures:**

Two metrics of prevalence and persistence of CRN and their adjusted coefficients in GEE with binomial family distribution and log link function controlling for socio-demographic and health characteristics.

**Results:**

A total of 5,990 quarterly surveys were completed by the 677 patients during the 30-month study period. Among the 677 patients, 250 (37%) were men, 591 (87%) were African American, and 288 (42%) were Medicare-Medicaid dual eligible. The unadjusted prevalence of CRN before and during the COVID-19 pandemic was 31.1% and 25.7% respectively (p = 0.02 by Chi-squared test), and persistent CRN rates were 12.1% and 9.7% respectively (p = 0.17 by Chi-squared test). The adjusted odds ratio of CRN prevalence during the pandemic compared to the pre-pandemic level was 0.75 (p<0.01), and 0.74 (p = 0.03) for persistent CRN in GEE estimations.

**Conclusion and relevance:**

There are coherent evidence of a reversal of CRN rates during the COVID-19 pandemic among this high-need, high-cost resource utilization Medicare population. Patients’ CRN behaviors may be responsive to exogenous impacts, and the behaviors changed in the same direction with similar magnitude in terms of prevalence (the extensive margin) and persistence (the intensive margin). More research is needed to advance the understanding of the driving forces behind patients’ behavioral changes and to identify factors that may be informative for reducing CRN in the long run.

## Introduction

Non-adherence to medication due to cost is a serious challenge in the US healthcare system, as one in four adults in the US have a difficult time affording their medications [1]. The COVID-19 pandemic has imposed an unprecedented burden on patients regarding seeking and remaining engaged in care. For high-need, high-cost resource utilization populations, such challenges may be particularly pronounced, as they require frequent care and many medications despite limited economic means. It is conceivable that economic pressures coupled with disrupted continuity of care may have affected their medical adherence. In the meantime, government policies to stabilize income and housing (including the Coronavirus Aid, Relief and Economic Security (CARES) Act, providing Americans with expanded unemployment insurance (UI) benefits if they’re out of work for pandemic-related reasons [2], and a temporary national moratorium on most evictions for nonpayment of rent [3]) may have helped patients at the very bottom of the economic ladder to ease the tension between their medical and other basic needs. In addition, states were prohibited from disenrolling people from Medicaid during the COVID pandemic, even if they no longer met the eligibility guidelines [4]. Furthermore, many patients in the high-need, high-cost Medicare population are younger than 65 as they become eligible for Medicare due to disability; they may or may not be eligible for Medicaid due to means tests and spouses or family members who are still in the labor force, or at the risk of losing it. Even for older patients among this population, because they are at the bottom of the economic ladder, their high price sensitivity due to budget constraint may affect CRN significantly. This is a vulnerable population whose medical care could be affected significantly by loss of subsidized housing, eviction, loss of unemployment benefits, or loss of Medicare-Medicaid dual eligibility in the absence of those federal policies protecting housing, unemployment benefits, and Medicaid eligibility, A knowledge and understanding of patients’ adherence to medical care during the pandemic is thus critical for evaluating the benefits of government policies and identifying factors external to the healthcare system that influence such behaviors. However, little is known about adherence behaviors during the pandemic, particularly among the high-need, high-cost resource utilization population.

We aimed to test the hypotheses that cost-related medication non-adherence (CRN)–patients not taking medications as prescribed due to cost–had changed for a 30-month period before and during the COVID-19 pandemic. We used a sample of Medicare patients at high risk of hospitalization and a novel data set of quarterly surveys of CRN with two metrics of prevalence and persistence to reflect two aspects of such behaviors [5]. These two metrics measure CRN behaviors on the extensive margin (i.e., any CRN behavior), and intensive margin (i.e., the persistence in CRN) and hence reflect well the patients’ behavioral changes before and during COVID-19.

## Methods

We used a sample of Medicare patients at high risk of hospitalization, a high-need, high-cost resource utilization population, who were hospitalized at least once in the previous year or cared for at the emergency department at the time of enrolled into a randomized controlled trial to study the Comprehensive Care Physician (CCP) model, where the CCP integrated the physician services for ambulatory care and inpatient care for patients at high risk of hospitalizations with ongoing quarterly surveys of patients in an urban academic medical center [5, 6]. CRN questionnaire is an original part of the CCP study to advance the understanding of cost barriers to health care. The CCP study is a longitudinal study with the subjects followed up quarterly since enrollment, and follow-up is still ongoing. Medicare is a federal program providing health insurance to 65 million Americans aged 65 or older, under age 65 with certain disabilities, and people of all ages with End-Stage Renal Disease [7]. While the program is heavily subsidized, beneficiaries incur out-of-pocket payments for deductible, co-insurance, and premiums. In traditional Medicare, most beneficiaries’ spending does not exceed $5,000, but 4.5 million people spend over $5,000 per year out-of-pocket and 1.6 million beneficiaries spend $10,000 or more annually [8]. For context, the national average spending per person in Medicare was $15,300 in 2021 [9]. We estimated that the patients in the CCP study incurred 300–400% of the average annual health cost for Medicare beneficiaries during the year before the enrollment, and they represent the high-need, high-cost resource utilization population well because many are older adults with multiple chronic conditions, and experience multiple functional limitations. The high-need, high-cost population has been identified as an urgent national healthcare priority [10]. Another common feature of such a high-need, high-cost population is its high mortality rate due to high disease burden; this sample is no exception. Hence, to represent this population sample fully during the COVID pandemic, the additional inclusion criteria for this secondary data analysis included patients being alive on January 1, 2020, during which month the first COVID-19 case was reported in the US [11], and having completed at least one quarterly follow-up survey during the first quarter of 2020, to enable comparison between pre- and during pandemic periods with inclusion of those who were deceased during the pandemic. While at least 1 follow-up survey before the pandemic was not explicitly required, given the long follow-up before the pandemic, all subjects have had at least 1 survey before the pandemic. The study sample was followed quarterly in surveys from October 1, 2018, to March 31, 2021, a period of 15 months before the COVID-19 pandemic (October 1, 2018- December 31, 2019) and 15 months during (January 1, 2020-March 31, 2021). One novel advantage of such a data set is the quarterly survey of CRN, as repeated assessments enable measuring the persistence of CRN behaviors. The national data sets with CRN are predominantly annual surveys [12] which do not allow for a longitudinal study of persistence of CRN over time. In addition, research has shown that respondents often forget events from the first half of the year on an annual survey [13]. By tracking the quarterly survey over time, we were able to measure the persistence of CRN before and during the pandemic and thus gauge the intensity of CRN behaviors, whose change is little known in the literature.

Questionnaires on CRN were administered in the surveys by asking during the past three months, have you ever done the following due to cost: 1) not fill or refill a prescription, 2) delay filling a prescription, 3) skip doses, or 4) take smaller doses to make medication last longer. CRN then was categorized as 1 if the patients reported any of these 4, and 0 if none.

We developed two metrics of CRN: prevalence for any CRN reported during pre- and during pandemic periods, and persistence of CRN if CRN was reported on more than half of the 5 quarterly surveys during each 15-month period.^4^ Prevalence was measured by any CRN reported in any survey during the 15-month pre-pandemic and pandemic periods, while persistence was measured by whether patients more often than not reported CRN during the two periods [5]. For example, for patients who were followed up for 5 surveys before or during the pandemic, persistence in CRN was noted if patients reported CRN 3 or more times. If patients were deceased or dropped out during the study period, persistence was noted if CRN was reported on 3 or more out of 4 surveys or 2 or more out of 3. We first compared the rates of prevalence and persistence of CRN before and during the pandemic period using Chi-square tests. We also described the trajectory of CRN rates by quarter from October 1, 2018, to March 31, 2021, graphically. We linked our survey data to Medicare enrollment and Vital Statistics files to ascertain death information, as death is a major reason for sample attrition and a key factor for shortened follow-up. The Medicare Vital Statistics file was updated up to July 14, 2022; hence we were able to remove those who were deceased before January 1, 2020, from the denominator of the analysis as those patients did not have any comparable CRN rates during the pandemic.

To further ascertain the population-adjusted change in prevalence and persistence of CRN between the pre-pandemic and pandemic periods, we conducted analyses using Generalized Estimating Equation (GEE) with binomial family distribution, log link function, and exchangeable correlation structure to address unmeasured correlation among the subjects [14, 15]. controlling for confounders including age, gender, race, ethnicity, educational attainment, and health literacy at the baseline. The age variable, with a cut-off of 65 years on January 1, 2019, was derived from the date of birth from the Medicare Vital Statistics file. Research shows a different risk profile for CRN for those under 65 as many more in that age group have disabilities [16], hence such a cut-off reflects disability status and socio-economic status (receiving Social Security Disability Income) rather than merely a biological age. The indicator variables for race were derived from the intake survey of the study, as self-reported race is considered the gold standard [17]. Educational attainment and health literacy were also derived from the intake survey [18]. We included a set of comorbidity indicators for cancer, cardiovascular disease, depression, diabetes, and kidney disease, self-reported in the follow-up surveys and summed up by the study periods, as these conditions have been found to be risk factors for CRN [12]. We also included an indicator variable for Medicare-Medicaid dual eligibility at the time of enrollment, as Medicare beneficiaries with dual eligibility are sicker patients with fewer economic resources but more generous insurance coverage. Research has shown that patients with dual eligibility have a different risk profile for CRN [19], and understanding the role of dual eligibility in this context is important for medical practitioners and policy makers. Since this data set came from a study originally intended for developing the CCP model in a randomized controlled trial, we also added one indictor variable for the study arm to generate population-adjusted rates for comparison before and during the pandemic. The analysis was conducted using Stata Statistical Software version 14 (StataCorp LP, College Station, TX).

This report follows the Strengthening the Reporting of Observational Studies in Epidemiology (STROBE) reporting guideline for cohort studies. The study was approved by our Institutional Review Board. The patient consents were obtained through written consent.

## Results

The original CCP study has a sample size of 2000 subjects with 1000 subjects in each study arm and enrollment took place between November 2012 and June 2016. After removing 12 subjects who were randomized in error or withdrawn by patients from the study, we were able to link 1976 (99.4%) subjects out of 1988 successfully to the Medicare enrollment file, with correct linkage ascertained through matching birthdates between our internal survey and Medicare Vital Statistics. Among those 1976 subjects, 721 (36.5%) were ascertained to be deceased before January 1, 2020, through Medicare Vital Statistics, hence were excluded from the study. Those patients were deceased before the pandemic and thus a pre- and during pandemic comparison is not possible. Among the remaining 1,255 subjects, 677 (52.8%) were followed up before and during the pandemic, with at least one follow-up survey during the first quarter of 2020, and the rest (578 subjects, 46.1%) were lost to follow-up between November 2012, and January 1, 2020. Among those 677 patients, 56 (8.3%) died between January 1, 2020, and March 31, 2021, and 358 (52.9%) came from the intervention arm and 319 from the standard care of the original CCP study.

A total of 5,990 quarterly surveys were completed by the 677 patients during the 30-month study period; on average, 4.6 (s.d. 0.93) surveys per person were completed pre-pandemic, and 4.3 (s.d. 1.3) surveys per person were completed during the pandemic.

Table 1 shows the demographics and health characteristics for the 677 study subjects. Among the 677 patients, 250 (37%) were men, 591 (87%) African American, 288 (42%) Medicare-Medicaid dual eligible.

**Table 1:** Patient socio-demographic and health characteristics.

Total Patients: N (%)	677 (100)
Age as of January 1, 2019	
<65 years: N (%)	235 (34.7)
65 or older: N (%)	442 (65.3)
Sex	
Female: N (%)	427 (63.1)
Race	
White: N (%)	43 (6.4)
Black: N (%)	588 (86.9)
Other race: N (%)	14 (2.1)
Don’t know/refused: N (%)	32 (4.7)
Hispanic ethnicity: N (%)	29 (4.3)
Medicare-Medicaid	
Dual Eligibility: N (%)	288 (42.5)
Education	
< High school	135 (19.9)
High school graduate: N (%)	183 (27.0)
Some college or junior college: N (%)	197 (29.1)
College graduate: N (%)	147 (21.7)
Don’t know/refused: N (%)	15 (2.2)
Self-reported health conditions	
Cancer: N (%)	50 (7.4)
Cardiovascular Disease: N (%)	222 (32.8)
Depression: N (%)	158 (23.3)
Diabetes: N (%)	227 (33.5)
Kidney Disease: N (%)	121 (17.9)
Health literacy	
Extremely or quite confident filling out medical forms: N (%)	445 (65.7)
Somewhat/a little bit/not at all filling out medical forms: N (%)	232 (34.3)
Don’t know/refused: N (%)	8 (1.2)
Study Group	
Comprehensive Care: N (%)	358 (52.9)
Standard of Care: N (%)	319 (47.1)

**Fig 1:**
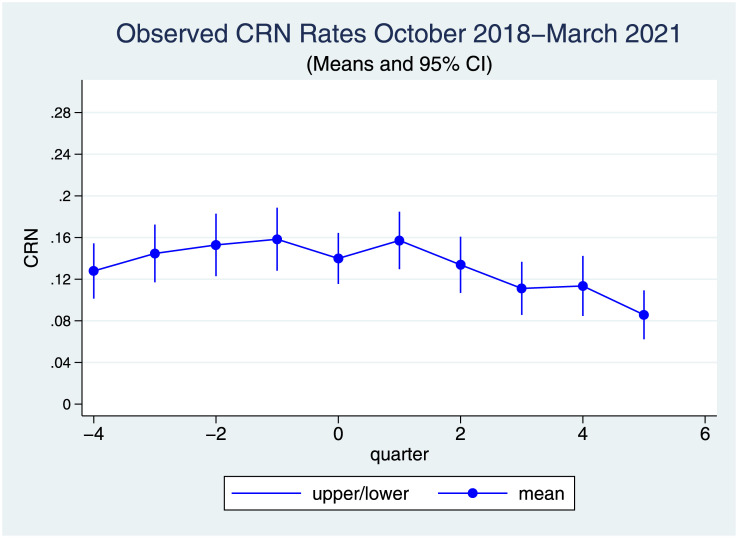
Observed CRN rates October 2018-March 2021. Quarters indicating 5 pre-pandemic quarters (October 2018-December 2019) and 5 quarters during pandemic (January 2020 –March 2021) with the last quarter of 2019 as 0.

The observed CRN rates by quarter are illustrated in Fig 1. There was an upward trend in quarterly CRN prevalence before the pandemic, and then the trend reversed during the pandemic.

Table 2 shows the observed (unadjusted) prevalence and persistence of CRN before and during the pandemic. The prevalence of CRN before and during the COVID-19 pandemic was 31.1% and 25.7% respectively (p = 0.02 by Chi-squared test), and persistent CRN rates were 12.1% and 9.7% respectively (p = 0.17 by Chi-squared test).

**Table 2:** Observed CRN prevalence and persistence pre- and during COVID-19 pandemic (N = 677).

	Pre-COVID-19 (10/1/2018-12/31/2019) N (%)	During COVID-19 (1/1/2020-3/31/2021)	P-value by Chi-square Test
Prevalence of CRN (Any CRN)	210 (31.0%)	174 (25.7%)	0.03
Persistence of CRN (More often than not reporting CRN)	81 (12.0%)	65 (9.6%)	0.16

Table 3 shows the correlates and their associated adjusted coefficients from the Generalized Estimating Equation analyses of CRN prevalence and persistence, respectively. Age under 65 was consistently a significant risk factor (AOR = 1.88, 2.38, p<0.01, respectively) compared to those 65 or older. Those with cancer were more likely to report CRN in persistence (AOR = 2.36, p<0.01); those with depression were more likely to report CRN in prevalence (AOR = 1.49, p = 0.01). The adjusted odd ratio of CRN prevalence during the pandemic compared to the pre-pandemic level was 0.75 (p<0.01), and 0.74 (p = 0.03) for persistent CRN in GEE estimations.

**Table 3:** Adjusted odds ratios of prevalence and persistence of CRN during pandemic and correlates.

	CRN Prevalence				CRN Persistence			
	Odds Ratio	P>z	[95% Conf.	Interval]	Odds Ratio	P>z	[95% Conf.	Interval]
During pandemic (ref: pre-pandemic)	0.75	<0.01	0.62	0.91	0.74	0.03	0.57	0.97
Age < 65	1.88	<0.01	1.37	2.57	2.39	<0.01	1.50	3.82
Male (ref: female)	1.01	0.93	0.75	1.37	0.85	0.49	0.54	1.35
Race: Black	Reference	Reference	Reference	Reference	Reference	Reference	Reference	Reference
White	0.83	0.55	0.44	1.55	0.88	0.79	0.35	2.21
Race: other	2.12	0.11	0.84	5.36	2.65	0.09	0.85	8.24
Race: don’t know or refused	0.99	0.97	0.46	2.12	0.47	0.22	0.14	1.56
Hispanic ethnicity	1.67	0.21	0.75	3.70	3.80	0.01	1.38	10.42
Education: (ref: college)	Reference	Reference	Reference	Reference	Reference	Reference	Reference	Reference
< High school	0.83	0.42	0.53	1.30	0.48	0.05	0.23	1.00
High school	1.03	0.88	0.71	1.50	0.74	0.28	0.42	1.29
Post-graduate	0.80	0.30	0.53	1.21	0.84	0.57	0.47	1.51
Education: don’t know or refused	0.43	0.16	0.13	1.41	0.26	0.29	0.02	3.11
Limited health literacy	0.83	0.27	0.60	1.16	0.94	0.82	0.57	1.56
Medicare-Medicaid dual eligibility	0.85	0.32	0.63	1.17	1.02	0.92	0.64	1.63
Cancer	1.07	0.78	0.69	1.66	2.36	<0.01	1.36	4.08
Diabetes	0.92	0.58	0.67	1.24	1.10	0.69	0.70	1.72
Depression	1.49	0.01	1.12	1.97	1.21	0.34	0.82	1.80
Cardiovascular condition	1.34	0.05	1.00	1.81	1.24	0.34	0.80	1.92
Kidney condition	0.72	0.08	0.49	1.04	0.57	0.05	0.32	1.00
Study arm: Intervention	1.35	0.04	1.01	1.81	1.18	0.45	0.76	1.83

Results from the Generalized Estimating Equation analyses with log link function, binomial family function, and exchangeable correlation structure, for CRN prevalence and persistence, respectively.

## Discussion

To our knowledge, this is the first study on cost-related medication non-adherence longitudinally following a sample of high-need, high-cost resource utilization Medicare patients before and during the pandemic. We found that despite increased economic pressure and disrupted medical care, there was a reverse of trend in CRN rates, with significant declining in prevalence and persistence in CRN during the pandemic when compared to the pre-pandemic period.

The significant declining CRN prevalence may be reflective of economic relief efforts by the government [2–4] and increased awareness of the risk of not taking medicine during the pandemic. This suggested that patients’ CRN behaviors are responsive to exogenous impact at the extensive margin, and further research to tease out the impact of economic relief and change in perceived risk may provide further insight into how to influence CRN at the social and health policy level. The longitudinal aspects of CRN behaviors were not well understood in the literature, and this study brings new insight through a large-scale pandemic that social and health policies may work in tandem to reduce CRN in both extensive margin (prevalence) and intensive margin (persistence) with far-reaching impact on patients’ economic well-being.

In the meantime, little is known about the change in persistent CRN behaviors in the literature, and this study reveals that those who had persistent CRN behaviors could also be influenced by exogenous impacts of similar magnitude. This is important because the CRN behaviors at the extensive margin (prevalence) and intensive margin (persistence) may have different tenacity. More research is needed to understand patients’ responsiveness to the social and health policy to reduce CRN in the long run.

In this study, we found the those under 65 had significantly higher likelihood of reporting CRN in both prevalence and persistence. These patients became eligible for Medicare due to disability, and their spouses and family members were likely still in the labor force and hence may not be eligible for Medicaid due to means test or at the risk of losing Medicaid coverage. Their struggle with medical needs is most likely to be affected by the absence of protection for housing, unemployment benefits, and Medicaid eligibility. More research is needed to understand their CRN trajectory once such protection is removed.

Furthermore, this study demonstrated that relieving economic pressure could be conducive to reducing CRN. Another example of possible policy action is to limit out-of-pocket payments for Medicare patients., The insight gained from this study may be useful for understanding the impact of a ceiling of economic risk or a floor of protection on continuity of care in terms of implementing the Inflation Reduction Act in the following years.

In this study, we did not look specifically into the different patterns with which subgroups changed CRN behaviors–for example, staying with, moving away from, and newly reporting CRN. These are complex transition patterns, and our modest sample size does not allow us to examine the subgroups in such detail with these many covariates. Nevertheless, knowing that CRN behaviors may change at both extensive margin of prevalence and intensive margin of persistence under exogenous impacts underlines the promising prospect that policy intervention through economic relief may reduce CRN behaviors and hence save significant sums of downstream costs, and effective intervention to reduce CRN may also be devised by influencing patients’ perceived risk.

This study is limited in that the sample is a predominantly African American population in an urban setting; hence it is not representative of the general population and may not be generalizable to the entire Medicare population. However, the high-need, high-cost resource utilization patient population is an urgent policy priority with a high risk of CRN. National data sets often do not have a large enough sample size to investigate such a subpopulation, and particularly lack longitudinal follow-up to allow the measurement of persistent behavior. The insight gained from this study may be informative for further research on patient behaviors in this important population and may guide the evaluation of national economic policies. This study is also limited in that the original study cohort was recruited during 2012–2016 and followed up to March of 2021, during which time many were deceased. The sample attrition may have a “survival effect” on those who remained in the study, as mortality was the main driver for sample attrition (721 subjects from the original sample died before January 1, 2020, and hence were excluded from this study). However, the sample size of the intervention and standard care groups in the study were comparable, reflecting the original feature of the enrollment. While we included that about 8.3% of the sample subjects died during the study period, their trajectory of CRN is unknown in the literature. The lack of power did not allow us to do a stratified study on those who were deceased. In addition, while the US CDC first alerted clinicians on January 8, 2020, to be on the look-out for patients with respiratory symptoms and a history of travel to Wuhan, China,^10^ the public awareness of COVID-19 gradually increased over time. As a result, patients’ response to COVID-19 could have become stronger as they learned more about it from their clinicians and mass media. Such a time gradient is beyond the scope of this study and our modest sample size does not allow us to further segment the response by timing, but the importance of timing of information may warrant careful examination to better understand patients’ behavioral changes. Finally, there is also possibility that medication regimen during the pandemic was changed for some patients to reduce the opportunity of transmitting COVID. The financial consequence for such a change is uncertain, and future research should examine the likelihood of change in clinical care during pandemic for those high-need, high-resource utilization patients and its financial consequence to direct medical costs. Finally, COVID might have impacted CRN through many pathways; for example, while patients may have reduced or delayed their in-person office visits, telemedicine may have facilitated remote access to care and hence offset such an impact. The net result is largely an empirical question, and the investigation of such a pathway is beyond the scope of the current study. While we incorporated time-varying health variables in our GEE analysis, the study is limited by a relatively short follow-up period. Future study should be directed to understanding the long-term CRN trajectory [20].

## Conclusion

In summary, we presented coherent evidence of a reversal of CRN rates during the COVID-19 pandemic among this high-need, high-cost resource utilization Medicare population. It demonstrates that patients’ CRN behaviors may be responsive to exogenous impacts, and that behaviors changed in the same direction with similar magnitude in terms of prevalence (the extensive margin) and persistence (the intensive margin). More research is critically needed to advance understanding of the driving forces behind patients’ behavioral changes and to identify factors that may be informative for reducing CRN in the long run.

## References

[1] Henry J. Kaiser Family Foundation. *Poll*: *Nearly 1 in 4 Americans Taking Prescription Drugs Say It’s Difficult to Afford Their Medicines*, *Including Larger Shares Among Those with Health Issues*, *with Low Incomes and Nearing Medicare Age*: *Bi-Partisan Majorities Support Range of Policy Changes Aimed at Lowering Drug Costs*. 2019. Accessed June 25, 2019. https://www.kff.org/health-costs/press-release/poll-nearly-1-in-4-americans-taking-prescription-drugs-say-its-difficult-to-afford-medicines-including-larger-shares-with-low-incomes/

[2] Garner J. New COVID-19 Unemployment Benefits: Answering Common Questions. *US Department of Labor Blog*. January 11, 2021. Accessed October 8, 2021. https://blog.dol.gov/2021/01/11/unemployment-benefits-answering-common-questions.

[3] National Low Income Housing Coalition. *The Centers for Disease Control and Prevention (CDC) Took Unprecedented Action by Issuing a Temporary National Moratorium on Most Evictions for Nonpayment of Rent to Help Prevent the Spread of Coronavirus*. Accessed October 8, 2021. https://nlihc.org/coronavirus-and-housing-homelessness/national-eviction-moratorium.

[4] The Kaiser Family Foundation. State Policy Choices Are Likely to Affect the Extent of Medicaid Enrollment Declines During the Unwinding Period. https://www.kff.org/medicaid/issue-brief/state-policy-choices-are-likely-to-affect-the-extent-of-medicaid-enrollment-declines-during-the-unwinding-period/. Accessed on May 16, 2023.

[5] De Avila JL, Meltzer DO, Zhang JX. Prevalence and persistence of cost-related medication nonadherence among Medicare beneficiaries at high risk of hospitalization. *JAMA Netw Open*. 2021;4(3):e210498. doi: 10.1001/jamanetworkopen.2021.0498 33656528 PMC7930921

[6] Meltzer DO, Ruhnke GW. Redesigning care for patients at increased hospitalization risk: the Comprehensive Care Physician model. *Health Aff (Millwood)*. 2014 May;33(5):770–7. doi: 10.1377/hlthaff.2014.0072 . 24799573 PMC4331340

[7] Centers for Medicare & Medicaid Services. Medicare Program—General Information. https://www.cms.gov/Medicare/Medicare-General-Information/MedicareGenInfo. Accessed on January 1, 2023.

[8] Gangopadhyaya A, Holahan J, Garrett B, and Shartzer A. Adding an Out-of-Pocket Spending Limit to Traditional Medicare. Urban Institute, 2022.

[9] Kaiser Family Foundation. The Facts About Medicare Spending. https://www.kff.org/interactive/medicare-spending/. Accessed on January 1, 2023.

[10] Blumenthal D, Chernof B, Fulmer T, Lumpkin J, Selberg J. Caring for high-need, high-cost patients—an urgent priority. *N Engl J Med*. 2016;375(10):909–911. doi: 10.1056/NEJMp1608511 27602661

[11] First travel-related case of 2019 novel coronavirus detected in United States. News Release. Centers for Disease Control and Prevention; January 21, 2020. Accessed October 8, 2021. https://www.cdc.gov/media/releases/2020/p0121-novel-coronavirus-travel-case.html.

[12] Briesacher BA, Gurwitz JH, Soumerai SB. Patients at-risk for cost-related medication nonadherence: a review of the literature. *J Gen Intern Med*. 2007;22(6):864–871. doi: 10.1007/s11606-007-0180-x 17410403 PMC2219866

[13] Horvath FW. Forgotten unemployment: recall bias in retrospective data. US Bureau of Labor Statistics. Accessed October 28, 2021. https://www.bls.gov/opub/mlr/1982/03/rpt3full.pdf.

[14] Liang KY and Zeger S. Longitudinal data analysis using generalized linear models". Biometrika. 1986;73 (1): 13–22. doi: 10.1093/biomet/73.1.13

[15] James H and Hilbe J. Generalized Estimating Equations. London: Chapman and Hall/CRC. 2003. ISBN 978-1-58488-307-4.

[16] Soumerai SB, Pierre-Jacques M, Zhang F, Ross-Degnan D, Adams AS, Gurwitz J, et al. Cost-related medication nonadherence among elderly and disabled medicare beneficiaries: a national survey 1 year before the medicare drug benefit. *Arch Intern Med*. 2006 Sep 25;166(17):1829–35. doi: 10.1001/archinte.166.17.1829 . 17000938

[17] Jarrín OF, Nyandege AN, Grafova IB, Dong X, Lin H. Validity of race and ethnicity codes in Medicare administrative data compared with gold-standard self-reported race collected during routine home health care visits. *Med Care*. 2020;58(1):e1–e8. doi: 10.1097/MLR.0000000000001216 31688554 PMC6904433

[18] Chew LD, Griffin JM, Partin MR, et al. Validation of screening questions for limited health literacy in a large VA outpatient population. *J Gen Intern Med*. 2008;23(5):561–566. doi: 10.1007/s11606-008-0520-5 18335281 PMC2324160

[19] Zhang JX, Meltzer DO. The high cost-related medication non-adherence rate among Medicare-Medicaid dual-eligible diabetes patients. *J Health Med Econ*. 2016;2:13. 28795170 PMC5546751

[20] Zhang J, Bhaumik D, Meltzer D. Decreasing rates of cost-related medication non-adherence by age advancement among American generational cohorts 2004–2014: a longitudinal study. *BMJ Open*. 2022 May 6;12(5):e051480. doi: 10.1136/bmjopen-2021-051480 . 35523499 PMC9083426

